# NHLBI perspectives on the growth of heart, lung, blood and sleep conditions in Africa: global and domestic insights, challenges and opportunities

**DOI:** 10.5830/CVJA-2015-044

**Published:** 2015

**Authors:** Gary H Gibbons, Nakela L Cook, Uchechukwu KA Sampson, George A Mensah

**Affiliations:** National Heart, Lung, and Blood Institute (NHLBI), and National Institutes of Health (NIH), Bethesda, USA; National Heart, Lung, and Blood Institute (NHLBI), and National Institutes of Health (NIH), Bethesda, USA; Center for Translation Research and Implementation Science (CTRIS), National Heart, Lung, and Blood Institute, and National Institutes of Health, Bethesda, USA; Center for Translation Research and Implementation Science (CTRIS), National Heart, Lung, and Blood Institute, and National Institutes of Health, Bethesda, USA

**Keywords:** health inequities, cardiovascular diseases, lung diseases, sickle cell disease, sleep disorders, biomedical research

## Abstract

‘Of all forms of inequity, injustice in healthcare is the most shocking and inhumane’

Dr Martin Luther King, Jr, March 25, 1966, 2nd National Convention of the Medical Committee for Human Rights

The mission of the National Heart, Lung, and Blood Institute (NHLBI) centres on global leadership in research, training and education aimed at promoting the prevention and treatment of heart, lung, blood and sleep (HLBS) disorders, and thereby enhance the pursuit of a healthy, long and fulfilling existence by all individuals.^1^ The global horizon of this mission reflects an appreciation of the collective destiny shared by all humanity

Martin Luther King envisioned a world that is increasingly inter-dependent, a world in which we are all part of a ‘beloved community’, where every life matters. In this vision of a ‘beloved community’ he also said that ‘…injustice anywhere is a threat to justice everywhere…’. If indeed health inequity is an injustice; then it is incumbent upon the global public health community to recognise the threat that health inequities pose to the entire human family all around the world.

The advent of globalisation and the attendant shrinkage of the degrees of human separation compel us to work collectively to address the critical challenges that stand in the way of ideal health everywhere. The NHLBI is committed to working with health researchers from sub-Saharan Africa (SSA) and across the globe to build this future together.^2^

In this research endeavour, the NHLBI’s strategy for successful stewardship at national and global levels rests on several enduring principles, which include: valuing and supporting investigator-initiated fundamental discovery science; maintaining a balanced, cross-disciplinary research portfolio; supporting implementation science that empowers patients and enables partners to apply knowledge that improves the health of the nation; training and nurturing a diverse new generation of leaders in science; engaging key thought-leaders to collectively identify and pursue high-yield opportunities that will advance the field; valuing the health of all communities; and innovating an evidence-based elimination of health inequities in the United States and around the globe. The continued prioritisation of these enduring principles is supported by the observed trends in HLBS conditions and other chronic non-communicable diseases at the global and domestic fronts.

## Relevant trends

Several recent trends in the burden of heart, lung and blood diseases provide illustrative examples of why our collective effort in this endeavour is necessary. For example, in 2010 the global age-standardised disability-adjusted life years (DALYs) per 100 000 population associated with sickle cell disorders in SSA was 281.16 (CI: 196.70–368.44), substantially in excess of the estimated 77.86 (CI: 58.01–98.96) for other developing regions, and compared to 80.09 (CI: 60.00–102.40) globally.^3^ Piel and colleagues project that the numbers of newborns with sickle cell anaemia (SCA) globally will increase from 305 800 (238 400–398 800) in 2010 to 404 200 (242 500–657 600) by the year 2050, and that Nigeria and the Democratic Republic of Congo will remain the countries most in need of policies for the management of SCA.^4^

Additionally, the report indicates that the implementation of large-scale universal screening can save the lives of up to 9 806 000 (6 745 800–14 232 700) newborns with SCA globally, 85% (81–88%) of whom will be born in SSA. Similarly, we can achieve significant reduction in mortality and prolong the lives of 5.3 million newborns with SCA if we implement basic health interventions such as prenatal diagnosis, penicillin prophylaxis and vaccination for children under five years of age.^4^

Obesity is a major public health problem with significant impact on global morbidity, mortality and economic development. In developed regions, the age-standardised deaths per 100 000 population associated with high body mass index (BMI) decreased from 77.42 (CI: 65.37–89.31) in 1990 to 68.23 (CI: 59.09–77.06) in 2010, however in SSA the rates increased from 21.01 (CI: 14.73–28.28) to 37.85 (CI: 29.80–46.70) during the same period.5 Likewise, in developed regions, the age-standardised DALYs associated with high BMI decreased from 1978.99 (CI: 1660.99–2299.62) in 1990 to 1914.45 (CI: 1649.36–2190.76) in 2010. On the contrary, the rates in SSA increased from 623.03 (431.17–842.82) in 1990 to 1 141.23 (CI: 889.99–1 412.23) in 2010.^5^

Global trends reflecting challenges and opportunities in achieving HLBS health include those existing on the domestic front. The 2013 US National Healthcare Disparities report demonstrates that there is ample room for improvement. The risk-adjusted in-patient mortality rate for heart attack hospital admissions fell significantly between 2001 and 2010 for each racial/ethnic and area income group; however residents of the lowest area income quartile had higher in-patient mortality rates than residents of the highest area income group in five of the 10 years evaluated.^6^

In 2008, Hispanic men and women were less likely to receive blood pressure measurements compared with their white counterparts. Furthermore, although vaccination for pneumococcal pneumonia is a cost-effective strategy for reducing illness, death and disparities associated with pneumonia and influenza, blacks and Asians were less likely than whites, and Hispanics were less likely than non-Hispanics to receive immunization, among the elderly who reported ever receiving pneumococcal vaccination.

Similarly, among hospital patients, age 50 years and above with pneumonia who received influenza immunisation status assessment or provision, black, Hispanic, American Indians/Alaskan natives (AI/AN) and Asian patients were less likely than white patients to receive influenza immunisation status assessment or provision. Also, among long-term nursing home residents, black, AI/AN, multiple-race and Hispanic residents were less likely than white residents to receive both influenza and pneumococcal immunisation.

From 2003 to 2010, the percentage of people with current asthma who reported taking preventative asthma medicine daily or almost daily fell from 29.6 to 26.5%. In five of eight years, blacks compared with whites, and poor and low-income people compared with high-income people, were less likely to take daily preventative asthma medicine.

## New and evolving insights

The above noted trends occur in an era where new insights are reshaping our understanding of the complexities of disease mechanisms, while prompting us to contemplate transformative ways to prevent and pre-empt the burden of HLBS conditions. In the wake of recent reports, the intimate and intricate interplay between social and biological systems in the pathobiology of HLBS conditions is increasingly appreciated.

In the context of obesity, a central risk factor in the domain of HLBS disorders, the report by Christakis and Fowler evokes the notion of the social contagion of disease.^7^ They demonstrated that obesity appears to spread though social ties, and therefore network phenomena may be relevant to the biological and behavioural trait of obesity. Simply stated, a socio-ecological construct underpins trends in disease evolution, which implies that it matters where we live, learn, work and play, and that culture, religion, war, food desserts and unhealthy diets all play into the determinants of HLBS conditions. The philosophy of social contagion of disease has profound implications for disease intervention, because if we are to successfully tackle inequities in HLBS conditions, we have to embrace the understanding that we are dealing with a complex multi-level problem that warrants a systems science intervention approach.^8^,^9^

The mechanisms by which socio-behavioural and biological factors interact in the pathobiology of disease are now increasingly palpable and not just a figment inspired by epidemiological studies. Recent evidence suggests a relationship between longterm dietary patterns and gut microbial enterotypes,^10^ that a link exists between intestinal microbial metabolism and cardiovascular risk,^11^ and that the microbiota of the gut is a potentially novel target for atherosclerosis prevention and treatment.^12^ In addition to the emerging evidence for the impact of diet on human health via modulation of the composition of gut microbiome, we are reminded that complex genetic interplay attend disease mechanisms, and therefore can inform our approaches for risk prediction, pharmacogenomics and new therapies, particularly in the context of genomic-based medicine strategies to reduce health inequities.^13^,^14^

## Challenges and opportunities

The above insights should inspire the deployment of systems science in search of major proximal targets in the socioecological model that could lead to a transformative impact on HLBS conditions. Despite the challenge of austere budgets for biomedical research, we remain committed to making a transformative impact by maintaining a balanced portfolio to reflect the strategic goals of the NHLBI, which include promoting the understanding of human health and disease, translating basic research into preventative and therapeutic interventions, and developing a biomedical workforce with the requisite set of skills for advancing HLBS research. There also exists the challenge of maintaining a balance between achieving these goals and encouraging creativity. These challenges converge to significantly impact on our decisions and approaches for tackling health inequities at home or abroad to advance the unfinished business of maximising the public health impact.

In the focus on addressing health inequities, we often fail to recognise the extraordinary resilience and resourcefulness of people working to improve health in high-risk communities. As we work collectively to overcome the challenge of reducing global health inequities, we should recall the admonition of Theodore Roosevelt: ‘Do what you can, with what you have, where you are’. This is an opportune moment in history to tap in to the resilience and resourcefulness of this ‘beloved’ global community in order to create a collective future in which population health systems serve to promote the health of the entire human family, and we bend the curves of health inequities at the domestic and global level.

The optimum approach for addressing this question is to catalyse systems science, which will entail the employment of community health knowledge networks; a diverse pool of cross-disciplinary investigators; and leveraging NHLBI study platforms such as health systems clinical or population-based cohorts to optimise the prediction, pre-emption and treatment of HBLS conditions using new tools and platforms. Herein, outstanding possibilities attend the confluence of advances in genomics research and technology, imaging, informatics, computational modelling, stem cell research, nanotechnology and bioengineering, and collaborative knowledge-intervention networks. These new tools and platforms provide impetus for us to consider transformative questions.

What if we could develop new paradigms for citizen-enabled community health and next-generation cardiovascular disease prevention, i.e. networks and systems of learning communities that continually seek wellness and innovate to improve health? What if we were able to realise precision and personalised medicine to prevent and pre-empt the burden of cardiovascular, lung and blood diseases? What if we could eliminate stroke and cognitive impairment in persons living with SCD by providing access to the benefits of chronic blood transfusions,^15^ or better still, what if we could find ways to up-regulate modifier genes or identify new vasculopathy targets that could completely transform the landscape of cerebrovascular outcomes in SCD patients?

While we seek novel approaches, including systems science, new tools and platforms and genomics, we would be remiss if we forgot implementation research, which is paramount for advancing adherence to best practices for health promotion: diets rich in fruits and vegetables, physical activity, tobacco avoidance or smoking cessation; and the prevention and treatment of cardinal risk factors such as high blood pressure, dyslipidaemia and diabetes, which are central to HLBS conditions. In this regard, can we imagine the hypothetical scenario wherein we optimally disseminate and implement evidence-based ‘best buy’ approaches, with the resultant improvement in the social wellbeing and productivity of many around the world?

## Conclusion

The importance of disease prevention and treatment, as well as the social significance of science, call for collaborative partnerships in response to adverse trends in HLBS conditions and related disorders, both at the domestic and global fronts. It is gratifying to observe the evolution of such a collaborative network under the umbrella of the H3Africa initiative. The SSA region as well as the USA will benefit immensely from such partnership models in an effort to rise to the emerging challenges posed by the growth of HLBS diseases and risk factors.

More importantly, vertical integration of efforts across continents may prove to be very helpful and synergistic as we seek to discover new targets or transformative approaches for maximising population-level impact and consequently reduce health inequities. For instance, lessons learned from novel approaches for improving outcomes among SCD patients in SSA may be very helpful for SCD patients in the domestic USA. Therefore there are benefits that can come full circle when we address life’s most persistent and urgent question, as eloquently posed by Dr Martin Luther King Jr: ‘What are we doing for others?’ This is the era to create a collective future, because the concept of collective destinies could not be more palpable than it is now.
